# Supplementary material for: Thermodynamic Study of the Solubility of Triclocarban in Polyethylene Glycol 200 + Water Cosolvent Mixtures at Different Temperatures
Source: Molecules. 2025 Jun 17;30(12):2631. doi: 10.3390/molecules30122631 (PMC12195668; doi:10.3390/molecules30122631)
Supplement: Supplementary file 1 [file molecules-30-02631-s001.zip › molecules-3687118-supplementary.pdf]

Calibration curve of Triclcarban

λ_{Peak}: 265 nm
Solvent: ethanol obtained
Laboratory: Universidad Cooperativa de Colombia

1 Project Summary

Reply 1		Reply 2		Reply 3	
$\mu g_{TCC}/g_{soln}$	**Absorbance**	$\mu g_{TCC}/g_{soln}$	**Absorbance**	$\mu g_{TCC}/g_{soln}$	**Absorbance**
10.021	1.227	10.031	1.238	10.034	1.241
8.020	0.978	8.123	0.987	8.058	0.984
6.021	0.738	6.012	0.725	6.012	0.729
4.022	0.501	4.102	0.510	4.012	0.499
2.012	0.265	2.102	0.270	2.012	0.261
1.021	0.140	0.981	0.135	0.999	0.121

Table S1: Absorbance data of ethanolic solutions with different concentrations of Triclocarban

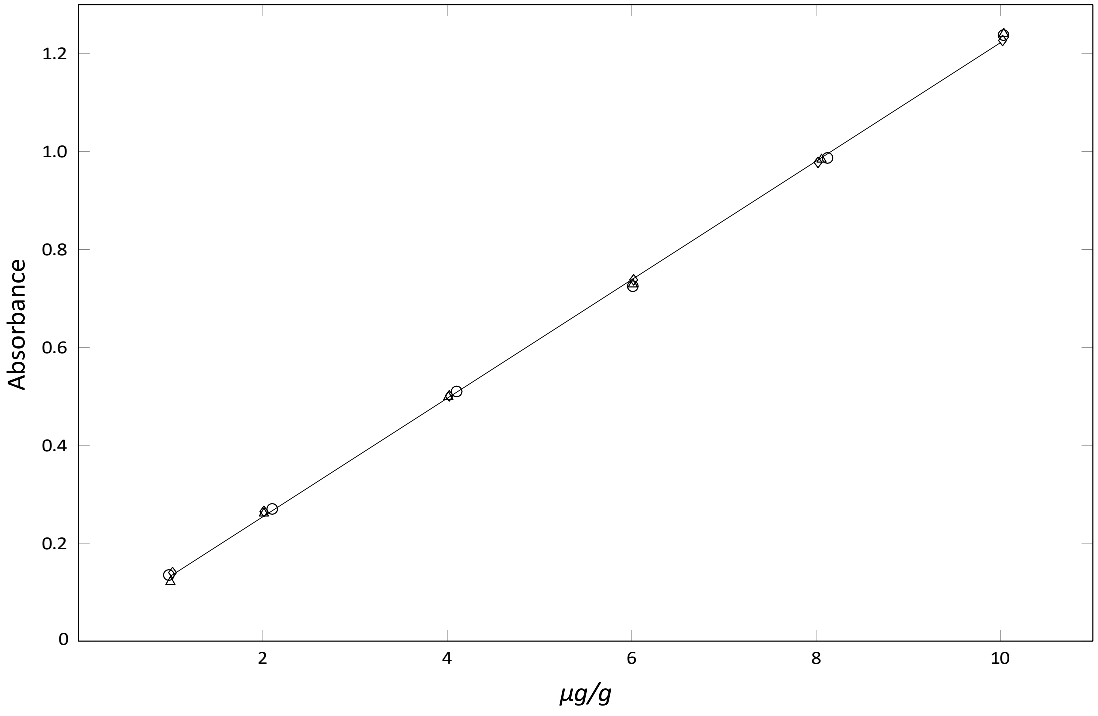

Figure S1: Calibration curve of triclocarban in absolute ethanol obtained at a wavelength of 265 nm.

Calibration curve equation

$$C = 8.255A - 0.0986 \quad (1)$$

where C is concentration in $\mu g/g$ and A is an absorbance

Multiple R.	0.999793856
R squared.	0.999587755
Adjusted R square	0.999561989
Standard Error of the regression	0.008329394
Observations	18

Table S2: Regression statistics

	Degrees of freedom	Sum of Squares	Mean squared error	F	Significance F
Regression	1	2.691610217	2.691610217	38795.85348	1.63841E-28
Residual	16	0.001110061	6.93788E-05		
Total	17	2.692720278			
	Coefficients	Standard Error	t Stat	p-value	
Intercept	0.0117494	0.0037524	3.1311779	0.0064447	
slope	0.1211314	0.0006150	196.96663	1.63841E-28	

Table S3: ANOVA

The detection limit (DL) may be expressed as:

$$DL = \frac{3.3\sigma}{S} \tag{2}$$

where σ = the standard deviation of the response, and S = the slope of the calibration curve Walfish (2006).

The quantitation limit (QL) may be expressed as:

$$QL = \frac{10\sigma}{S} \tag{3}$$

where σ = the standard deviation of the response, and S = the slope of the calibration curve
Thus DL and QL are 0.102 $\mu g_{TCC}/g_{soln}$ and 0.310 respectively $\mu g_{TCC}/g_{soln}$ Walfish (2006).

References

Walfish, S. Analytical Methods: A Statistical Perspective on the ICH Q2A and Q2B Guidelines for Validation of Analytical Methods. *BioPharm Int.* **2006**, *19*, 40–45.
